# Food insecurity and social protection in Europe: Quasi-natural experiment of Europe's great recessions 2004–2012

**DOI:** 10.1016/j.ypmed.2016.05.010

**Published:** 2016-08

**Authors:** Rachel Loopstra, Aaron Reeves, Martin McKee, David Stuckler

**Affiliations:** a Department of Sociology, Oxford University, Manor Road Building, Manor Road, Oxford OX1 3UQ, UK; b International Inequalities Institute, London School of Economics and Political Science, Houghton Street, London WC2A 2AE, UK; c Faculty of Public Health and Policy, London School of Hygiene and Tropical Medicine, 15–17 Tavistock Place, London, WC1H 9SH, UK

**Keywords:** Food insecurity, Social protection, Recession

## Abstract

Food insecurity rose sharply in Europe after 2009, but marked variation exists across countries and over time. We test whether social protection programs protected people from food insecurity arising from economic hardship across Europe. Data on household food insecurity covering 21 EU countries from 2004 to 2012 were taken from Eurostat 2015 edition and the Organisation for Economic Cooperation and Development. Cross-national first difference models were used to evaluate how rising unemployment and declining wages related to changes in the prevalence of food insecurity and the role of social protection expenditure in modifying observed effects. Economic hardship was strongly associated with greater food insecurity. Each 1 percentage point rise in unemployment rates was associated with an estimated 0.29 percentage point rise in food insecurity (95% CI: 0.10 to 0.49). Similarly, each $1000 decreases in annual average wages was associated with a 0.62 percentage point increase in food insecurity (95% CI: 0.27 to 0.97). Greater social protection spending mitigated these risks. Each $1000 spent per capita reduced the associations of rising unemployment and declining wages with food insecurity by 0.05 percentage points (95% CI: − 0.10 to − 0.0007) and 0.10 (95% CI: − 0.18 to − 0.006), respectively. The estimated effects of economic hardship on food insecurity became insignificant when countries spent more than $10,000 per capita on social protection. Rising unemployment and falling wages are strong statistical determinants of increasing food insecurity, but at high levels of social protection, these associations could be prevented.

## Highlights

• Food insecurity significantly rose in Europe over 2009 to 2012.
• Rising unemployment and falling wages linked to rising food insecurity within countries.
• Estimated effects became insignificant where spending on social protection was high.
• Rising food insecurity not inevitable in the face of economic downturn.

## 1 Introduction

The Great Recessions across Europe have been accompanied by growing concern about food insecurity (García, 2013, RFI, 2014, Smith, 2013, Taylor-Robinson et al., 2013a). Household food insecurity is broadly defined as “the limited or uncertain availability of nutritionally adequate and safe food, or having to acquire foods in socially unacceptable ways” (Anderson, 1990). Across Europe, the number of people reporting being unable to afford a meal with a protein source every other day, the only surveillance measure of food insecurity in Europe, was declining over 2005 to 2009, falling from 12% of the EU-27 population to 8.7%. But in 2010, this trend reversed, with food insecurity rising to 10.9% in 2012 and remaining elevated in 2013. After 2010, on average, an estimated total of 13.5 million additional people were food insecure over 2011 to 2013 over and above the historical trend (Loopstra et al., 2015a). Given evidence that food insecurity places adults and children at elevated risk of eating diets of poor quality (Kirkpatrick and Tarasuk, 2008), with long term implications for health including diet-related chronic conditions, such as dyslipidemia and inflammation (Parker et al., 2010, Seligman et al., 2010, Shin et al., 2015, Tayie and Zizza, 2009), as well as more immediate risks such as nutrient inadequacy and iron deficiency (Skalicky et al., 2006), there is considerable cause for concern. Referring to the UK situation, the UK Faculty of Public Health argued that recent evidence of increasing malnutrition and hunger constituted a “public health emergency” (UK Faculty of Public Health, 2014).

Because there has been significant variation in the extent to which countries have been affected by the recent economic crisis and in how they have responded (Reeves et al., 2013, Reeves et al., 2014), Europe provides a quasi-experimental setting to study macroeconomic drivers of food insecurity and potential mitigating factors. Cross-state analyses in North America have shown increasing unemployment and poverty rates to be associated with rising food insecurity (Gundersen et al., 2014, Sriram and Tarasuk, 2015, Tapogna et al., 2004), but to our knowledge, these factors have not been examined across Europe. One potentially important preventive factor is the strength of social protection programs. These have been shown to help mitigate, for example, the impact of job loss on suicide (Stuckler and Basu, 2013), but have not been examined in relation to food insecurity in the European context. However, studies of various types of social protection programs, such as income benefits, from Canada and the United States, suggest that enhancements to elements of social security are associated with reduced food insecurity (Emery et al., 2013, Loopstra et al., 2015b). One recent example, from Ionescu-Ittu and colleagues, found that the introduction of a new child benefit in Canada was associated with a decline in food insecurity among eligible families, particularly those most vulnerable (Ionescu-Ittu et al., 2015).

Here, we test the hypothesis that rising economic hardship – particularly unemployment and wage declines – are associated with increased risk of food insecurity in Europe. Then, we evaluate whether differing types and degree of social security spending helped buffer populations facing these hardships from food insecurity.

## 2 Methods

### 2.1 Food insecurity data

To obtain country-level data on the prevalence of food insecurity, we used the indicator available in the Eurostat database (Eurostat, 2015a). These data are collected in the material deprivation module in the Survey of Income and Living Conditions (EU-SILC). Specifically, in this survey, the household reference person is asked “Can your household afford a meal with meat, chicken, fish (or vegetarian equivalent) every second day?” This indicator is used to capture food poverty in Ireland (Carney and Maitre, 2012) and to denote food-related material deprivation in the EU (Eurostat, 2015b). As food-based dietary guidelines across European countries recommend at least 1–2 servings of meat, chicken or fish (or alternative protein sources) every day (European Food Information Council, 2015) and the measure indicates a lack of financial resources to acquire one essential component of a nutritionally adequate diet, this indicator is aligned with the definition of household food insecurity (Anderson, 1990). Of note is that it does not specify duration of exposure nor capture multiple dimensions of food insecurity, such as hunger or insecure access to sufficient quantities of food captured, as are captured by the Household Food Security Survey Module (Tarasuk et al., 2014), but to our knowledge it is the only available annual comparative indicator of food insecurity across EU countries.

### 2.2 Economic hardship data

We collected available data on GDP per capita and unemployment rates on 21 countries, covering years 2004–2012, from Eurostat (Eurostat, 2015a). These were linked to data on real wages from OECD (OECD, 2015). All macroeconomic data were denominated in constant standard international dollars per capita adjusted for purchasing power parity (PPP).

### 2.3 Social protection data

Data on spending on social protection were collected from Eurostat (Eurostat, 2015a). These cover spending on a range of welfare programs, involving both cash-transfers and in-kind support. These were unemployment insurance, income support for people with disabilities, spending on sickness, child benefit payments and paternity coverage, and public pensions and income support in old age. Box 1 further describes these categories.

### 2.4 Statistical analysis

First, we examined the association of changes in food insecurity with changes in unemployment and real wages using a first-difference model, which eliminates time-invariant characteristics which may differ across countries. Our model is as follows:

$$\Delta \;{\text{Food Insecurity}}_{\mathrm{it}}=\beta_{1}{\text{ΔUnemployment}}_{\mathrm{it}}+\beta_{2}{\text{ΔWages}}_{\mathrm{it}}+\beta_{3}{\text{ΔYear}}_{t}+\varepsilon_{\mathrm{it}}$$

Here, *i* is country and *t* is year. Δ denotes the annual percentage point change for unemployment and food insecurity, and the real change in annual average wages within countries. ε is the error term. In a subsequent model we adjust for the annual real change in GDP per capita.

Next, moving to our second hypothesis, we assess whether and to what extent social security spending modified the relationship between food insecurity and these indicators of economic hardship. To do so, we first examine the interaction terms for total social protection spending with the change in unemployment rates and change in annual wages, as follows:

$$\Delta \;{\text{Food Insecurity}}_{\mathrm{it}}=\beta_{4}{\text{ΔUnemployment}}_{\mathrm{it}}+\beta_{5}{\text{ΔWages}}_{\mathrm{it}}+\beta_{6}{\text{ΔYear}}_{t}+\beta_{7}\text{Social Protection}+\beta_{8}\text{ΔUnemployment}\times {\text{Social Protection}}_{\mathrm{it}}+\beta_{9}\text{ΔWages}\times {\text{Social Protection}}_{\mathrm{it}}+\varepsilon_{\mathrm{it}}$$

To illustrate how different country levels of spending impacted the associations of unemployment and wages with food insecurity, we used margins plots to plot the estimates for a 1 percentage point change in unemployment and a $1000 decline in annual wages across the range of social protection spending values observed in our sample.

Next, we conduct an exploratory analysis to observe which of alternative social protection programs are protective or whether effect modification is observed across all forms of social protection spending. To do so, in separate models, we examine the magnitude of the modifying effect of each category of social protection spending. For each category of social protection spending, a variable capturing the residual spending on social protection outside of the category of interest was calculated and included in the model to adjust for potential confounding due to spending in other areas. We examine if social protection spending on old-age benefits also has a modifying effect as a specificity check. We would expect spending in this category to have no effect on the relationships between unemployment and wages with food insecurity because this spending does not go directly toward the working-age population.

All models were estimated using STATA v13.0, and robust standard errors adjusted for clustering effects of countries are reported.

## 3 Results

### 3.1 Prevalence of food insecurity

**Fig. 1:**
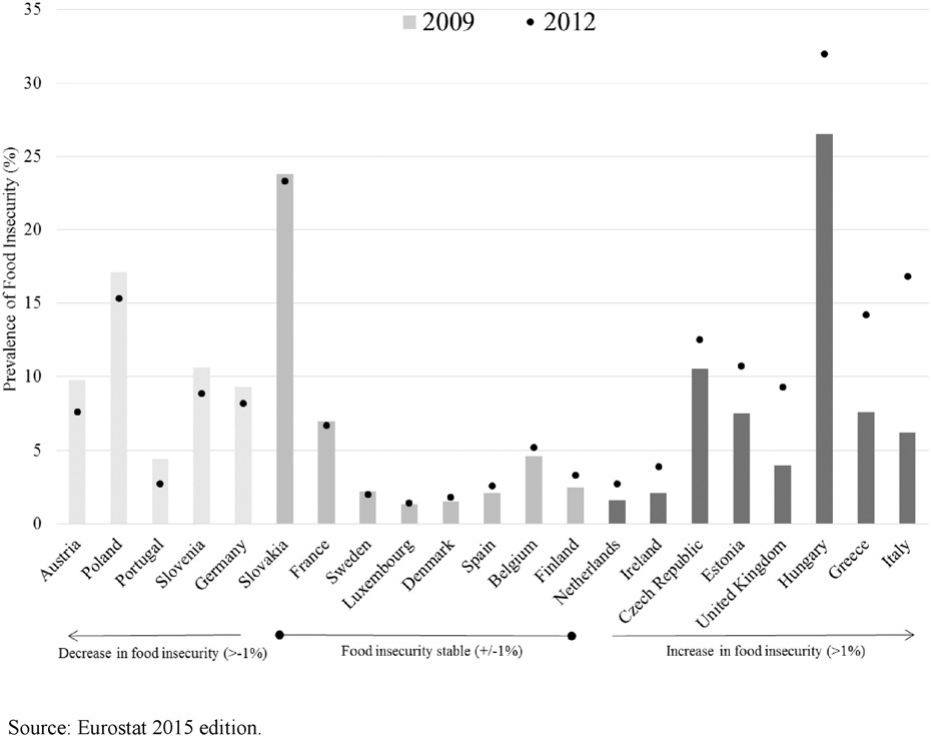
Prevalence of food insecurity in 2009 and 2012 in 21 EU countries.

As highlighted, Fig. 1 shows the variation in food insecurity both across EU countries and over time between 2009 and 2012, the period when food insecurity began to rise in the EU as a whole (Loopstra et al., 2015a). A handful of countries, such as Austria and Poland, experienced a decline in food insecurity over this period, but large rises in food insecurity were observed for the UK, Hungary, Green and Italy. There was no apparent patterning in the magnitude of change over this period by the level of food insecurity in countries in 2009.

### 3.2 Food insecurity and economic shocks

Table 1 shows the results of our cross-national statistical models quantifying the association of economic hardship with food insecurity. Each percentage point increase in unemployment is associated with an increase in food insecurity of 0.29 percentage points (95% CI: 0.10 to 0.49). Similarly, we observed that each $1000 decreases in annual wages was associated with a 0.62 percentage point increase in food insecurity (95% CI: 0.27 to 0.97). Adjusting for the change in GDP per capita strengthened the association between unemployment and food insecurity (β = 0.37; 95% CI: 0.14–0.60), but there was no residual association of GDP and incidence of food insecurity after adjusting for unemployment and wages (*p* = 0.10).

### 3.3 Preventive role of social protection

**Fig. 2:**
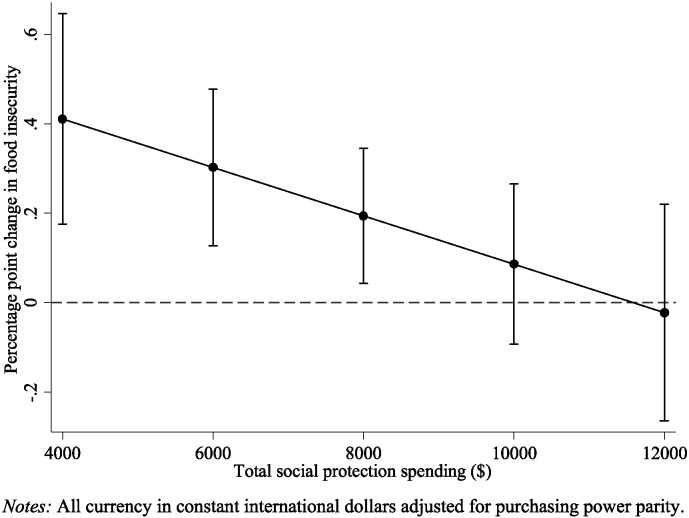
Predicted change in food insecurity associated with a 1 percentage point increase in unemployment rate by level of total social protection spending.

**Fig. 3:**
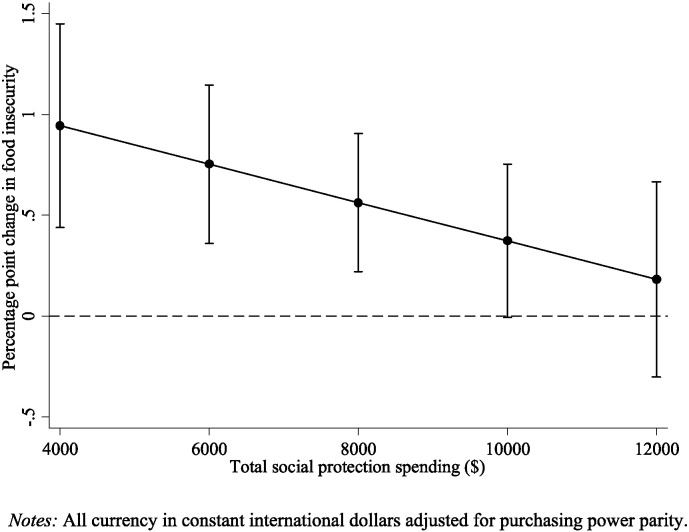
Predicted change in food insecurity associated with a $1000 decline in average annual wages by level of total social protection spending.

We observed that the impact of rising unemployment and falling wages on food insecurity were modified significantly by social protection spending (Model 3, Table 1). For every additional $1000 spent on social protection, the effect of rising unemployment on food insecurity was reduced by 0.05 (95% CI: − 0.10 to − 0.0007) percentage points and the effect of falling wages on food insecurity was reduced by 0.10 (95% CI: − 0.18 to − 0.006) percentage points. These relationships are illustrated in Fig. 2, Fig. 3. When social protection spending is around $4000 per capita, as found in the Czech Republic, Estonia, and Hungary, a one percentage point rise in unemployment is associated with a rise in food insecurity of 0.41 percentage points (95% CI: 0.18–0.65) and a fall in annual wages of $1000 is associated with a rise of 0.94 percentage points (95% CI: 0.44 to 1.45). In contrast, when social protection spending is above $10,000 per capita, as found in countries such as Sweden, Ireland, and Denmark, the effects of rising unemployment and falling wages on food insecurity become non-significant.

**Fig. 4:**
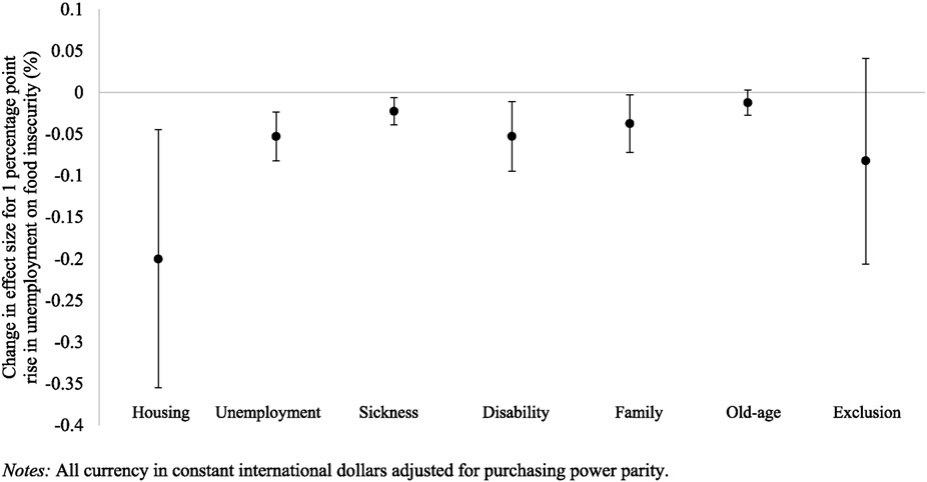
Reduction in effect of a one percentage point rise in unemployment rate on rise in food insecurity associated with an additional $100 spent on given social protection spending category.

Next we tested the modifying effects of different types of social protection on the relationships between economic hardship and incidence of food insecurity (Web Table A2). We found that, after accounting for residual social protection spending other than the category of interest, social protection spending on unemployment, housing, sickness, disability, and families all had significant modifying effects on the relationship between unemployment and food insecurity. Spending on old-age benefits had no impact on this relationship. Fig. 4 depicts the impact of different spending categories on the unemployment-food insecurity association. An additional $100 spent on housing reduced the incidence of food insecurity associated with a 1 percentage point rise in job loss by 0.20 percentage points (95% CI:− 0.35 to − 0.05). An additional $100 spent on unemployment insurance per capita reduced the incidence of food insecurity associated with a 1 percentage point rise in job loss by 0.05 percentage points (95% CI: − 0.08 to − 0.02).

**Fig. 5:**
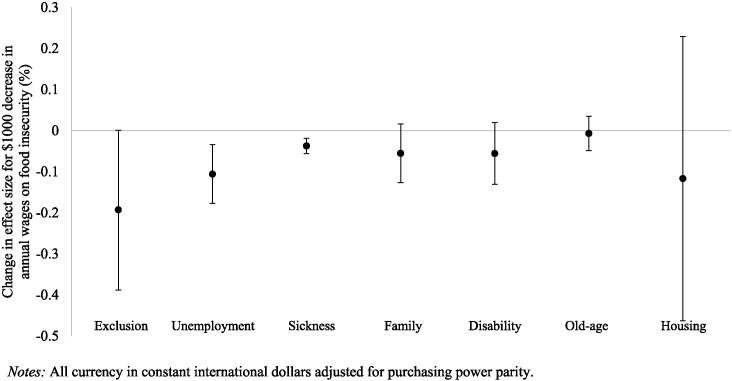
Reduction in effect of a $1000 decline in average annual wages on rise in food insecurity associated with an additional $100 spent in given social protection spending category.

Unemployment protection spending also reduced the association between falling wages and food insecurity (Web Table A2). As depicted in Fig. 5, for every additional $100 spend on unemployment insurance, the effect of a $1000 decline in average annual wages was reduced by 0.11 percentage points (95% CI: − 0.18 to − 0.03). There was also evidence that spending on sickness significantly modified the association, where every additional £100 spent per capita reduced the association for a $1000 decline in annual wages by 0.04 (95% CI: − 0.06 to − 0.02) percentage points. Spending on other areas did not significantly reduce the association between wages and food insecurity.

**Fig. 6:**
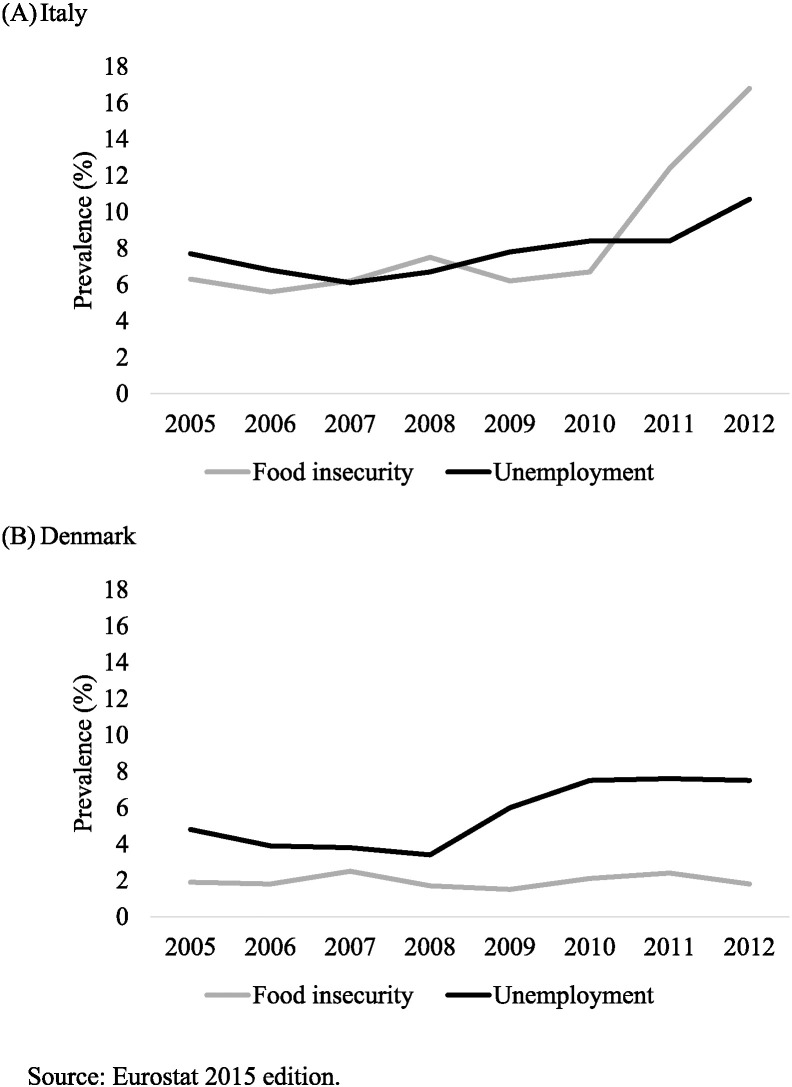
Trends in unemployment and food insecurity in Italy and Denmark.

To illustrate further we show two country examples of the impact of spending on unemployment insurance on the relationship between unemployment and food insecurity (Fig. 6). In Italy, where spending on social protection related to unemployment was, on average only $200 per capita per year between 2004 and 2012, food insecurity followed an almost parallel trajectory, even rising above increasing unemployment. In contrast, though Denmark, where the level of social protection spending on unemployment was, on average, over $600 per capita per year, despite also experiencing rising unemployment, food insecurity did not increase. Thus, this level of spending seems adequate to buffer the effects of rising unemployment.

### 3.4 Robustness checks

We performed a series of robustness checks to our model's specification and sample (Web Tables A3–A5). First we removed outliers based on standardized residuals >|2 | (n = 6). None of our results changed. Second, we included a time-dummy and observed comparable estimates. Lastly, we evaluated potential confounding from accelerating food price inflation between 2007 and 2012 in some countries in Europe. This had no significant association with food insecurity (β = 0.10; 95% CI: − 0.01 to 0.21) and did not change our findings. We also tested for potential autocorrelation of our model using a Cumby-Huizinga test for the first and second lag, and found no evidence of autocorrelation in the error structure of our model.

## 4 Discussion

In this paper we have shown that rising food insecurity within European countries was closely linked to rising unemployment and falling wages. Importantly, however, we showed that these features of economic downturn do not inevitably lead to households being unable to afford food. In countries where social protection spending has been high, rising unemployment did not lead to greater food insecurity. Similarly, where social protection spending was low, declining annual average wages were closely connected to increasing food insecurity. When we examined specific forms of social protection spending we found that welfare expenditure on unemployment buffered the effects of both unemployment and falling wages on rising food insecurity. Buffering effects were also observed with spending on housing, disability, sickness and family benefits and in-kind support.

Our findings align with cross-area studies conducted in the United States and Canada, which have shown rising unemployment rates and poverty rates to associate with trends in household food insecurity (Gundersen et al., 2014, Sriram and Tarasuk, 2015, Tapogna et al., 2004). To our knowledge, no studies have examined average wage rates, specifically, but our findings highlight the importance of adequate earned incomes for food security.

The protective role of social protection in buffering households from food insecurity arising from economic downturn has, to our knowledge, not been examined in Europe. Household-level studies from North America have demonstrated how the introduction of income- and in-kind interventions aimed at improving household incomes and reducing other household costs, such as child benefit payments, social housing provision, and prescription drug coverage, are associated with reductions in food insecurity (Loopstra et al., 2015b, Ionescu-Ittu et al., 2015). Social protection spending likely modifies the association between economic hardship and food insecurity through two mechanisms. First, these programs provide income support in the face of unemployment or low incomes. Income losses and unemployment are key triggers for household transition into food insecurity (Loopstra and Tarasuk, 2013, Huang et al., 2015), and these results suggest that income transfers play an important role in buffering these shocks to household income. Second, when faced with reduced incomes, households often have to make trade-offs between essential expenses such as food, utilities, housing, and medical expenses (Heflin, 2006, Frank et al., 2006). Our observation that social protection spending on housing and health modifies the association between economic hardship and food insecurity suggests that, where provision of housing and healthcare are assured, households face less need to prioritize these expenses over food, allowing them to maintain household food spending in the face of reduced incomes.

The data used in this study have certain limitations. The measure of food insecurity in Eurostat comes from the EU-SILC, a representative repeated cross-sectional survey conducted in EU member states. As with any observational study, selection effects and sampling error may lead to spurious changes in country-level estimates from year to year. The consistency of trends, both across Europe, and within countries, however, suggest that these data indicate real rises in food insecurity in the European population, which align with reports of increasing use of food aid (García, 2013, RFI, 2014, Taylor-Robinson et al., 2013a). This survey contains only this one indicator of household difficulty affording food, but to our knowledge, it is the only indicator of food insecurity available across EU countries and across time. It is important to note that household food insecurity has been defined more broadly and can manifest in different ways, over time, and in severity (Wunderlich and Norwood, 2006). Thus, using only one limited measure likely understates the full burden of food insecurity in Europe, where people may compromise their diets in other ways, such as by having smaller or less frequent meals, trading down foods for those of lower quality, or relying on charitable food assistance. They also may experience more severe manifestations, such as going without food for a whole day (Tarasuk et al., 2014, Coleman-Jensen et al., 2012). The measure also did not specify time of exposure, so it is unclear whether respondents would indicate only present circumstances or report based on their circumstances in the past year. Temporal differences could have made it harder to detect relationships with changes in the unemployment rate and annual average wages. The estimates obtained from this survey are also likely conservative because populations most vulnerable to food insecurity, such as people who are homeless, are not included, and homelessness has also been rising over this period (European Federation of National Organisations Working with the Homeless (FEANTSA), 2012). The adoption of regular monitoring of food insecurity using an in-depth measure in detailed population surveys in European countries, with boosts and appropriate sampling methods to include adequate numbers from hard to reach groups would enable better elucidation of both household- and policy drivers of this problem.

We were limited in the types of country-level variables we could investigate due to the few countries with available data. Social and cultural factors, such as reliance on family support, could mitigate the effects of unemployment and declining wages in some countries; however, our analytic focus on within-country annual change removes between-country differences and it is likely that these types of factors were relatively stable within countries over this period, therefore would not explain the within-country changes we observed. We were unable to capture other changes in public policy and public spending that may have impacted rising food insecurity over this time. For example, medical expenses may influence the amount of money households can spend on food (Tarasuk et al., 2013). Thus, policy changes such as increases in co-payments and user fees, such as those implemented in Greece (Reeves et al., 2015), may have increased food insecurity among low income individuals now having to struggle with these costs. Other factors that may impact household budgets for food include rising housing costs, job insecurity, and transportation costs.

We also note that the categories of social protection spending are broad and that we could not evaluate the effectiveness of different types of programs within these categories. Heterogeneity in the mix of programs provided by different countries could explain why, for some categories such as housing and social exclusion, our estimates of interactions lacked precision, as different programs could have different preventive effects. Disentangling the impact of specific aspects of social protection programs would further enhance understanding of how food insecurity can be prevented and mitigated. We note we were also unable to specifically identify spending on food and nutrition programs such as nutrition-support programs for vulnerable groups, such as pregnant and breastfeeding mothers and low-income school children, that exist in some EU countries (Lucas et al., 2015). These merit dedicated investigation to see if they can smooth food consumption during economic hardship.

Future research should focus on implementing monitoring of household food insecurity using a more comprehensive measurement tool such as the USDA Household Food Security Survey Module, which is used to monitor food insecurity in the United States and Canada (Tarasuk et al., 2014, Coleman-Jensen et al., 2012). This would enable a better description of prevalence, chronicity, and severity of food insecurity, allow identification of vulnerable groups, and facilitate cross North American-EU comparisons. More research investigating the impacts of specific aspects of social protection programs, including nutrition programs, on prevention and intervention of food insecurity in Europe is also needed. Lastly, delineating the impacts of food insecurity on health and nutrition in Europe is another critical research direction.

## 5 Conclusion

The problems of hunger and malnutrition in Europe were a key motivation for development of social protection in Britain and elsewhere following the Second World War. In the UK, social protection was envisaged to ensure people were free from “want”, providing the assurance of basic necessities, such as food, in the face of economic uncertainty (Walker, 2005). Our findings highlight how social protection remains as important today, protecting people from food insecurity during a period of economic recession in Europe. Yet, they also raise concern that in countries where social protection spending has been low, or recently eroded due to austerity policies, unemployment and falling wages have closely tracked with rising food insecurity. These trends have been exemplified by the growth in food banks in some countries (Riches and Silvasti, 2014, Loopstra et al., 2015c). There is a risk that food insecurity will become a permanent feature of countries in places where social protection continues to undergo further spending reductions.

There have been numerous calls for public health professionals to speak out on the importance of social protection for the maintenance of basic needs, especially healthy and adequate diets (Taylor-Robinson et al., 2013b, Schrecker and Milne, 2015). Our study provides further evidence why public health professionals to take-up this call.

## Conflict of interest statement

The authors declare there is no conflict of interest.

## Transparency document

Transparency document. Image 1

## Figures and Tables

**Table 1:** Associations of food insecurity with job loss and wages declines across 21 EU countries and interaction with level of social protection spending.

	Percentage point change in food insecurity		
(1)	(2)	(3)
Per 1 percentage point increase in unemployment	0.29⁎⁎ (0.095)	0.37⁎⁎ (0.11)	0.63⁎⁎ (0.19)
Per $1000 increase in average annual wages	0.62⁎⁎ (0.17)	0.62⁎⁎ (0.17)	1.32⁎⁎ (0.39)
Per $100 rise in GDP per capita		0.022 (0.013)	0.015 (0.010)
Per $100 increase in social protection spending per capita			0.0088 (0.0043)
Unemployment⁎social protection spending			− 0.0054⁎ (0.0022)
Annual wages⁎social protection spending			− 0.0095⁎ (0.0043)
Country-years	166	166	166
*R*^2^	0.185	0.208	0.297

All currency in constant international dollars adjusted for purchasing power parity. Standard errors in parentheses. Models include average level of annual change in food insecurity over time (first difference of year), not shown.

⁎ *p* < 0.05.

⁎⁎ *p* < 0.01.

## Appendix A Supplementary data

Supplementary material. Image 2
