# Supplementary material for: Food insecurity and social protection in Europe: Quasi-natural experiment of Europe's great recessions 2004–2012
Source: Prev Med. 2016 Aug;89:44–50. doi: 10.1016/j.ypmed.2016.05.010 (PMC4976834; doi:10.1016/j.ypmed.2016.05.010)
Supplement: Supplementary file 2 — Supplementary material. [file mmc2.docx]

Web Appendix

Web Table A1 Descriptive statistics, 21 EU-countries, 2004-2012.

| Variable | Country-years | Mean | SD | Min | Max |
| --- | --- | --- | --- | --- | --- |
| Annual percentage point change food insecurity (%) | 166 | -0.25 | 1.75 | -6.90 | 5.70 |
| Annual change in GDP ($) | 166 | 5.37 | 13.69 | -57.53 | 51.45 |
| Annual percentage point change in unemployment (%) | 166 | 0.27 | 1.69 | -4.40 | 8.00 |
| Annual change in average wages ($) | 166 | 279.81 | 765.68 | -2473.00 | 3923.00 |
| Total social protection spending per capita ($) | 166 | 8283.91 | 3459.86 | 2058.29 | 16825.15 |

*Notes:* All currency in constant international dollars adjusted for purchasing power parity.

Web Table A2 Interactions of job loss and wage declines with social protection spending categories in association with change in food insecurity across 21 EU countries, 2004-2012.

|  | Percentage point change in food insecurity | | | | | | |
| --- | --- | --- | --- | --- | --- | --- | --- |
|  | (1) | (2) | (3) | (4) | (5) | (6) | (7) |
| Per 1 percentage point rise in unemployment | 0.32^**^ | 0.24^**^ | 0.23^**^ | 0.22^**^ | 0.24^**^ | 0.30^**^ | 0.31^**^ |
|  | (0.088) | (0.076) | (0.069) | (0.076) | (0.062) | (0.080) | (0.094) |
| Per $1000 decrease in average annual wages | 0.73^**^ | 0.61^**^ | 0.72^**^ | 0.54^**^ | 0.58^**^ | 0.64^**^ | 0.58^**^ |
|  | (0.20) | (0.18) | (0.18) | (0.16) | (0.18) | (0.17) | (0.15) |
| *Per $100 per capita increase in social protection spending on:* |  |  |  |  |  |  |  |
|  |  |  |  |  |  |  |  |
| Unemployment | 0.013 |  |  |  |  |  |  |
|  | (0.044) |  |  |  |  |  |  |
| Interaction with unemployment | -0.053^**^ |  |  |  |  |  |  |
|  | (0.014) |  |  |  |  |  |  |
| Interaction with wages | -0.11^**^ |  |  |  |  |  |  |
|  | (0.034) |  |  |  |  |  |  |
| Housing |  | 0.11 |  |  |  |  |  |
|  |  | (0.15) |  |  |  |  |  |
| Interaction with unemployment |  | -0.20^*^ |  |  |  |  |  |
|  |  | (0.074) |  |  |  |  |  |
| Interaction with wages |  | -0.12 |  |  |  |  |  |
|  |  | (0.17) |  |  |  |  |  |
| Sickness/healthcare benefits |  |  | 0.026 |  |  |  |  |
| Interaction with unemployment |  |  | (0.020) |  |  |  |  |
|  |  |  | -0.023^**^ |  |  |  |  |
| Interaction with wages |  |  | (0.0079) |  |  |  |  |
|  |  |  | -0.037^**^ |  |  |  |  |
| Disability benefits |  |  | (0.0088) |  |  |  |  |
|  |  |  |  | -0.066 |  |  |  |
| Interaction with unemployment |  |  |  | (0.032) |  |  |  |
|  |  |  |  | -0.053^*^ |  |  |  |
| Interaction with wages |  |  |  | (0.020) |  |  |  |
|  |  |  |  | -0.056 |  |  |  |
| Family/child benefits |  |  |  | (0.036) |  |  |  |
|  |  |  |  |  | -0.042 |  |  |
| Interaction with unemployment |  |  |  |  | (0.028) |  |  |
|  |  |  |  |  | -0.038^*^ |  |  |
| Interaction with wages |  |  |  |  | (0.017) |  |  |
|  |  |  |  |  | -0.055 |  |  |
|  |  |  |  |  | (0.034) |  |  |
| Social exclusion |  |  |  |  |  | -0.097 |  |
|  |  |  |  |  |  | (0.085) |  |
| Interaction with unemployment |  |  |  |  |  | -0.082 |  |
|  |  |  |  |  |  | (0.059) |  |
| Interaction with wages |  |  |  |  |  | -0.19 |  |
|  |  |  |  |  |  | (0.093) |  |
| Old-age benefits |  |  |  |  |  |  | 0.040 |
|  |  |  |  |  |  |  | (0.022) |
| Interaction with unemployment |  |  |  |  |  |  | -0.012 |
|  |  |  |  |  |  |  | (0.0072) |
| Interaction with wages |  |  |  |  |  |  | -0.0070 |
|  |  |  |  |  |  |  | (0.020) |
| Country-years | 166 | 165 | 166 | 166 | 166 | 166 | 166 |
| *R*^2^ | 0.31 | 0.28 | 0.32 | 0.31 | 0.31 | 0.30 | 0.32 |

*Notes:* All currency in constant international dollars adjusted for purchasing power parity. All models include first difference of year, GDP, residual spending on social protection outside of category of interest. Standard errors in parentheses. † p<0.10 ^*^ *p* < 0.05, ^**^ *p* < 0.01

Web Table A3 Associations of food insecurity with job loss and wages declines and across 21 EU countries with adjustment for year dummy.

|  | Percentage point change in food insecurity (%) |
| --- | --- |
| Per 1 percentage point rise in unemployment | 0.41^**^ |
|  | (0.14) |
| Per $1000 decrease in average annual wages | 0.31^*^ |
|  | (0.12) |
| Per $100 rise in GDP per capita | 0.0061 |
|  | (0.012) |
| Observations | 166 |
| *R*^2^ | 0.289 |

*Notes:* All currency in constant international dollars adjusted for purchasing power parity. Standard errors in parentheses. Model includes dummy variable for year not shown.

* *p* < 0.05, ** *p* < 0.01

Web Table A4 Associations of food insecurity with job loss and wages declines and across 21 EU countries with removal of outliers.

|  | Percentage point change in food insecurity |
| --- | --- |
| Per 1 percentage point rise in unemployment | 0.30^**^ |
|  | (0.087) |
| Per $1000 decrease in average annual wages | 0.47^**^ |
|  | (0.100) |
| Per $100 rise in GDP per capita | 0.015 |
|  | (0.0099) |
| Country-years | 155 |
| *R*^2^ | 0.239 |

*Notes:* All currency in constant international dollars adjusted for purchasing power parity. Standard errors in parentheses. Model includes average level of annual change in food insecurity over time (first difference of year), not shown. Model excludes observations with residuals > |mean + 2sd|.

* *p* < 0.05, ** *p* < 0.01

Web Table A5 Associations of food insecurity with job loss and wages declines and across 21 EU countries including adjustment for food price inflation.

|  | Percentage point change in food insecurity |
| --- | --- |
| Per 1 percentage point rise in unemployment | 0.44**  (0.13) |
| Per $1000 decrease in average annual wages | 0.57**  (0.18) |
| Per $100 rise in GDP per capita | 0.024  (0.014) |
| Annual percent change in food price inflation (%) | 0.097  (0.053) |
| Country-years | 166 |
| *R*^2^ | 0.230 |

*Notes:* All currency in constant international dollars adjusted for purchasing power parity. Standard errors in parentheses. Models include average level of annual change in food insecurity over time (first difference of year), not shown.

* *p* < 0.05, ** *p* < 0.01

Web Figure A1 Reduction in effect of a one percentage point rise in unemployment rate on rise in food insecurity associated with an additional $100 spent on given social protection spending category.


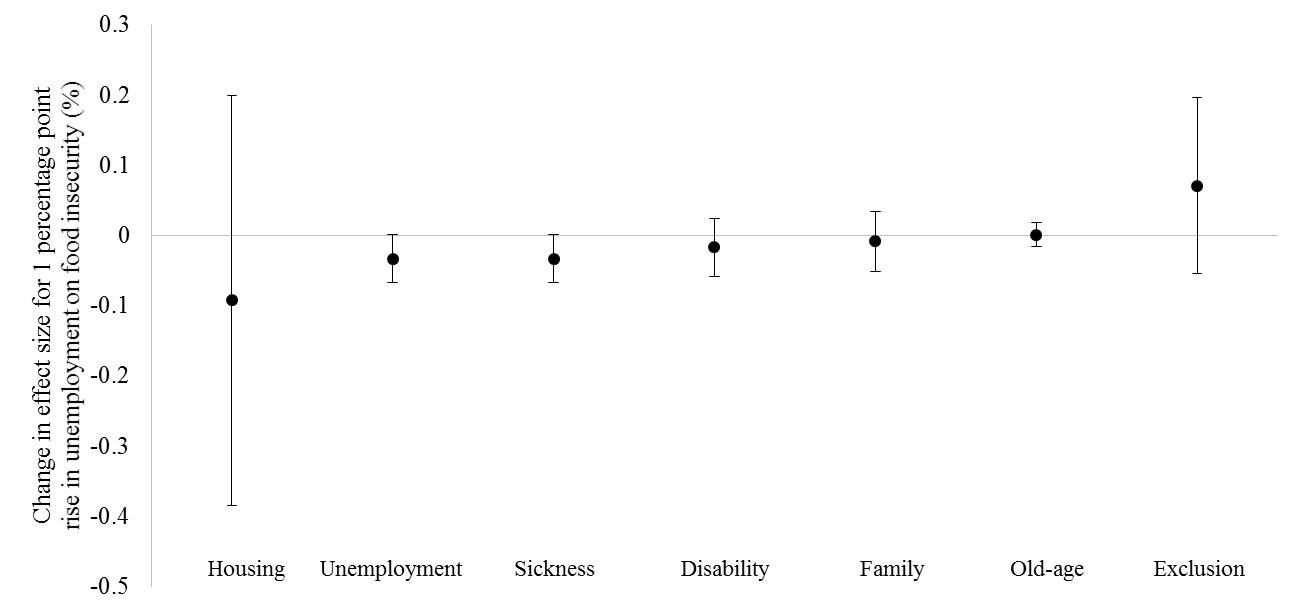


*Notes:* All currency in constant international dollars adjusted for purchasing power parity. Models include residual for social protection spending in other categories and respective interaction terms.

Web Figure A2 Reduction in effect of a $1000 decline in average annual wages on rise in food insecurity associated with an additional $100 spent in given social protection spending category.


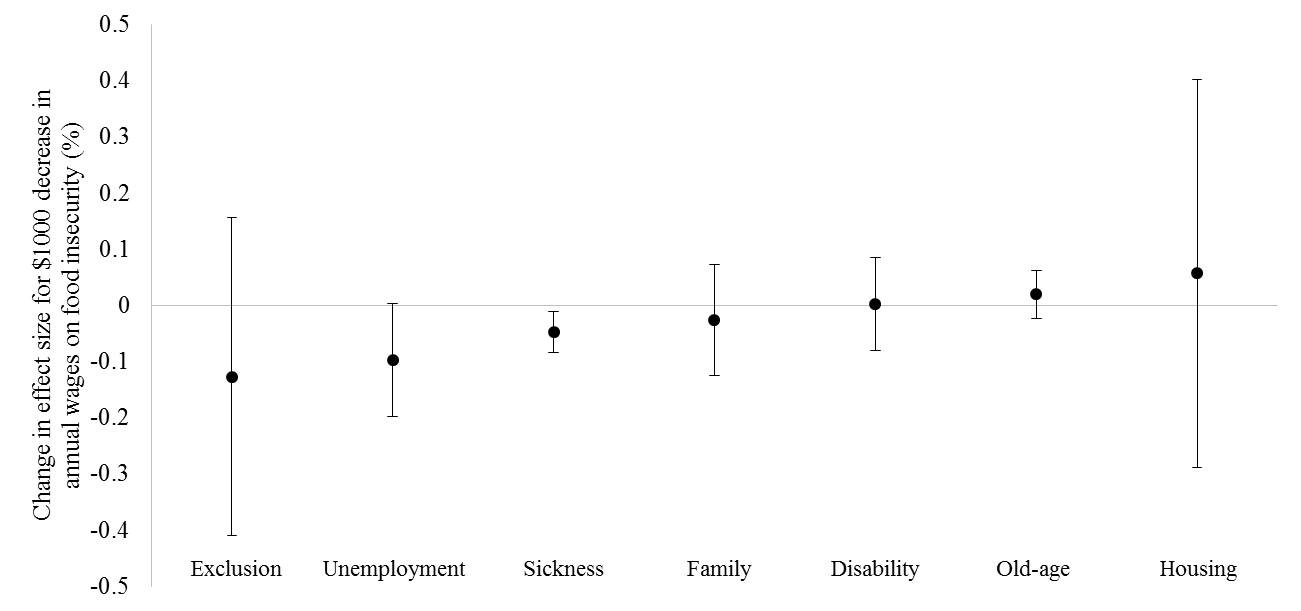


*Notes:* All currency in constant international dollars adjusted for purchasing power parity. Models include residual for social protection spending in other categories and respective interaction terms.
